# Prevalence and associated factors to cigarette smoking among school adolescents in Tunisia, 2021

**DOI:** 10.1192/j.eurpsy.2023.1834

**Published:** 2023-07-19

**Authors:** A. Silini, S. Rejaibi, M. Zid, S. Ben Youssef, N. Zoghlami, R. Mallekh, I. Ben Slema, N. Ben Salah, H. Aounallah-Skhiri

**Affiliations:** 1 Department of Epidemiology, National Institute of Health; 2 Nutrition Surveillance and Epidemiology department, SURVEN Research Laboratory; 3 Medical Faculty of Medicine, Tunis El Manar University; 4 Intensive Care Unit department, Center for Urgent Medical Assistance, Tunis, Tunisia

## Abstract

**Introduction:**

Tobacco use among youth is a real public health concern in most developing countries. To provide recent epidemiological data regarding tobacco use among this specific population, a national survey was conducted in Tunisia in 2021. We aimed to determine cigarette smoking prevalence in Tunisian adolescents and assess associated factors.

**Objectives:**

We aimed to determine cigarette smoking prevalence in Tunisian adolescents and assess associated factors.

**Methods:**

Data from the Mediterranean school survey on alcohol and other drugs (MedSPAD 2021) were used. Based on three-stage stratification sampling method, first and second grade high school students were enrolled. A self-administered standardized questionnaire was used and weighted prevalence estimates for cigarette smoking “at least once in a lifetime” were studied. Binary logistic regression model was used to assess associated factors and Adjusted Odds Ratios (AORs) were presented. The independent factors included were: sex, area of residence, private or public sector, alcohol and cannabis use, and being exposed to tobacco smoking in family and peer’s environment. Epidata and STATA software were used for data entry and statistical analysis, respectively.

**Results:**

Among 6.201 participants with a mean age of 16.8 years, 60.4% were girls; the prevalence of cigarette smoking was 24,75% 95% CI [23.24,26.32], significantly higher among boys (41.1% versus 14.2%, p<10^-3^). Univariate analysis revealed a significant difference in cigarette smoking by region (p-value< 10^-4^). The highest prevalence of cigarette smoking was observed in the capital city. Cigarettes were perceived as easily accessible by less than a third of the students (38.46% and 20.94% of boys and girls respectively, p=10^-4^). In multivariable analysis, the only independently associated factor to this behaviour was male sex (AOR=1.5[0.15 – 2.9], p-value=0.03).

**Conclusions:**

Our study revealed a high prevalence of smoking among students with male sex as an associated factor. Developing a healthy school environment and implementing school-based intervention programs are therefore, highly required.

**Disclosure of Interest:**

None Declared

